# Topical Application of Herbal Mixture Extract Inhibits Ovalbumin- or 2,4-Dinitrochlorobenzene-Induced Atopic Dermatitis

**DOI:** 10.1155/2012/545497

**Published:** 2012-12-05

**Authors:** Soon Re Kim, Han-Seok Choi, Hye Sook Seo, Youn Kyung Choi, Yong Cheol Shin, Seong-Gyu Ko

**Affiliations:** Department of Preventive Medicine, College of Oriental Medicine, Kyung Hee University, Seoul 130-701, Republic of Korea

## Abstract

KM110329 is four traditional herbal medicine mixtures with anti-inflammatory properties. Atopic dermatitis (AD) is an inflammatory skin disease associated with enhanced T-helper2 (Th2) lymphocyte response to allergens that results in elevated serum eosinophil and Immunoglobulin E (IgE) levels and leukocyte infiltration in atopic skin sites. In this study, we investigated the effect of topical application of KM110329 ethanol extract on the ovalbumin (OVA) or 2,4-dinitrochlorobenzene- (DNCB-) induced AD mouse models. For that purpose, we observed the effects of KM110329 on blood eosinophils, skin mast cells, production of serum IgE, and expression of cytokine mRNA in the atopic dermatitis skin lesions of OVA allergen- or DNCB-treated BALB/c mice. KM110329 significantly reduced blood eosinophils cell numbers in OVA or DNCB-treated BALB/c mice. Histological analyses demonstrated decreased mast cell count as well as dermal infiltration by inflammatory cells. In the skin lesions, mRNA expression of interleukine (IL)-4, IL-13, and IL-17 was inhibited by KM110329. KM110329 also suppressed the production of serum IgE level in both the OVA- and DNCB-induced atopic dermatitis model. Taken together, our results showed that topical application of KM110329 extracts exerts beneficial effects in AD symptoms, suggesting that KM110329 might be a useful candidate for the treatment of AD.

## 1. Introduction

Atopic dermatitis (AD) is a chronic inflammatory skin disease affecting about 10 million people in the world and its incidence is continuously increasing in westernized countries [1, 2]. The AD correlates with specific immune and inflammatory mechanisms. The general characteristics of AD include excessive infiltration of inflammatory cells such as lymphocytes, macrophages, and granulated mast cells into the skin lesions, eosinophilia in peripheral blood, and a high level of serum immunoglobulin E (IgE). 

The level of IgE is associated with severity of AD and contributed by abnormality of skin barrier, a key feature of AD. The functions of IgE in allergic inflammation suggest that IgE and IgE-mediated mast cell and eosinophils activation contribute to AD. IgE can sensitize mast cells in the skin culminating the production of inflammatory mediators, such as cytokines (IL-4, IL-5, IL-13, and tumor necrosis factor-alpha (TNF-*α*)), when cell-bound IgE is crosslinked by allergens. The cytokines IL-4 and IL-13 released by mast cells contribute to the Th2 response. TNF-*α* produced by macrophage also plays an important role in the acute phase of AD. The Th1 cytokines (IL-2 and interferon-gamma (IFN-*γ*)) play an important role in cell-mediated immunity and chronic inflammation. Although Th2 cells are dominant in the acute phase IFN-*γ* produced by Th1 cells is highly expressed and contributes to the pathogenesis in the chronic phase. IL-17 producing CD3+ T helper cells have crucial function in host defense, and dysregulated Th17 cell responses causes a variety of autoimmune and inflammatory disease [3]. The critical role of IL-17 in atopic dermatitis has recently been reported [4]. 

KM110329 is a mixture of four oriental herbal medicines (*Houttuynia cordata* Thunb, *Rehmannia glutinosa *Steud(Libosch), *Prunus yedoensis *Matsum, and *Rubus coreanus *Miq.). *Rehmannia glutinosa *has traditionally been used as an ingredient herb in East Asian medicine for the effects of hemostasis, activation of blood circulation, and improvement of kidney function [5]. Several studies indicated that *Rehmannia glutinosa* Libosch has antiallergy effects [6] and anti-inflammatory function [7–9]. *Houttuynia cordata *has been long used in traditional oriental medicine for the treatment of inflammation diseases. Also, several studies demonstrated that *Houttuynia cordata *has been associated with a broad range of pharmacological activities, including anti-inflammatory [10, 11], antiviral [12], and anticancer effects [13]. *Rubus coreanus*, is a type of red raspberry that grows wild in Korea, Japan and China. The fruit, known as “*Bokbunja*” in Korean, has been used in traditional oriental medicine for reducing the risk of diseases such as asthma and allergy. It is also known that *Rubus coreanus* has anti-inflammatory and antioxidative activities [14–16]. These collective observations indicate that KM110329 may be good candidate for control of AD and beneficial in the treatment of human allergic disorders. 

Therefore, in this study, we investigated whether 30% ethanol extract of KM110329 has useful activity in the treatment of AD using BALB/c male mice exposed to 2,4-dinitrochlorobenzene or BALB/c female mice exposed to ovalbumin. For our study, we measured eosinophils level in blood, histopathological changes including mast cells count, cytokine expression in skin tissue, and plasma IgE level in both model. 

## 2. Material and Method

### 2.1. Preparation of KM110329

Drugs were prepared by Hanpoong pharmaceutical (Jeon-ju, Korea) following good manufacturing practices (GMP) procedure. A ground powder (*Houttuynia cordata : Rubus coreanus : Rehmannia glutinosa : Prunus yedoensis* = 25 g : 25 g : 25 g : 25 g) with a mass of 100 g was extracted in 30% (V/V) ethanol by using an ultrasonicator (Branson, USA) for 30 min at room temperature. The alcohol extract was evaporated and then freeze-dried for 72 h (Freezedryer Japan). The powder from the extract was dissolved in distilled water.

### 2.2. Animals

Female BALB/c mice (aged 6 weeks) were used for OVA induced-AD model and male BALB/c mice (aged 6 weeks) were used for DNCB induced-AD model. The mice were purchased from Orient (Sung-nam, Korea) and randomized into three groups (Normal, OVA, KM110329 or Normal, DNCB, KM110329), each comprising of four mice. All mice were kept under pathogen-free environment and allowed free access to the diet and water. All procedures performed on the mice were approved by the animal care center of Kyung-Hee University (Approval No. KHUASP (SE)-2012-004). 

### 2.3. Sensitization and Treatment

**Figure 1:**
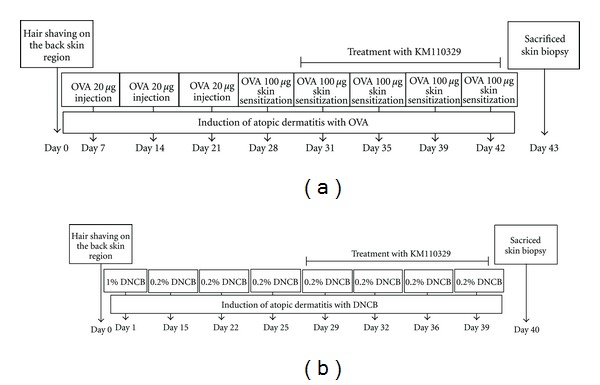
Protocol for induction of atopic dermatitis. Shaved dorsal regions of the mice were sensitized epicutaneously with OVA (a) or DNCB (b) solution. (a) Female BALB/c mice were intraperitioneally sensitized with OVA once a week for three weeks. After three weeks, the back of the mice was shaved with an electric razor and dermally challenged with 1 × 1 cm sterile patches containing OVA (100 *μ*g) for 3 weeks. KM110329 was applied to the skin during the second and third skin sensitization week together with OVA. (b) Male BALB/c mice were epicutaneously sensitized with 200 *μ*L of a 1% DNCB solution on day 1. Two weeks later, dermatitis was induced with 200 *μ*L of 0.2% DNCB solution at the intervals shown in figure. KM110329 was applied to the skin from the 3th week during sensitization together with DNCB.

The schematic experimental procedure is described in Figure 1. For establishment of OVA-induced AD model, female 7 weeks old BALB/c mice were intraperitoneally sensitized with 20 *μ*g of OVA once a week for three weeks. After three weeks, the back of the mice was shaved with an electric razor and dermally challenged with 1 × 1 cm sterile patches containing OVA (100 *μ*g) for 2 weeks. KM110329 was applied to the skin during the second and third skin sensitization week together with OVA. Patches were changed three times per week during sensitization (Figure 1(a)). For formation of DNCB-induced AD model, male 7 weeks old BALB/c mice were divided into three groups, each comprising of four mice. After shaving, mice back skin was painted dermally with 200 *μ*L of a 1% DNCB using 1 × 1 cm patches. Two weeks after sensitization, the back skin was challenged with 200 *μ*L of a 0.2% DNCB solution twice a week. This procedure was repeated for 4 weeks. KM110329 was applied to the skin from the 3th week during sensitization together with DNBC (Figure 1(b)). Mice were killed by CO_2_-inhalation, and samples were collected. 

### 2.4. Blood Analysis

After the final skin drug sensitization, whole blood samples were collected by cardiac puncture. The blood was placed in Vacutainer TM tubes containing EDTA (BD science, NJ, USA). Anticoagulated blood was processed to determine hematological parameters (lymphocytes, monocytes, eosinophils, and neutrophils) in a HEMAVET 950 hematology analyzer (Drew Scientific, Inc., Oxford, CT) in accordance to manufacturer's recommendation.

### 2.5. Histological Analysis

A portion of the skin biopsies were fixed in 4% paraformaldehyde (PFA) and embedded in Tissue-Tek optical cutting temperature (O.C.T) compound (Tissue-Tek, Sakura, AA Zoeterwoude, The Netherlands) on dry ice. Skin sections of 10 *μ*m were cut and stained with hematoxylin and eosin (H & E) for inflammatory cells or with toluidine blue for mast cells counts and examined under light microscopy (Olympus). Mast cells were counted in 10 parts of high-power fields (HPF) at 400x magnification.

### 2.6. Immunohistochemistry

Expression of CD3+ lymphocytes was detected by immnohistochemical analysis using specific antibody. Frozen sections of skin samples were cut into 10 *μ*m sections and fixed with cold 4% (PFA). Sections were immersed in 3% hydrogen peroxide for 20 min to eliminate endogenous peroxidase activity and then blocked with 5% bovine serum albumin in PBS for 1 h. Sections were incubated with mouse monoclonal CD3+ antibody overnight at 4°C and subsequently incubated with secondary biotinylated anti-rabbit IgG for 1 h at room temperature. Sections were treated with avidin-biotin HRP complex (Vectastatin ABC kit, Vector Labs, CA, USA) for 30 min at 4°C and stained with Diaminobenzidine tetrachloride (DAB) as the substrate. The slides were mounted with an aqueous mounting solution (DAKO, Glostrup, Denmark) and cover-slipped. All the sections were analyzed using an Olympus microscope and images were captured using a digital video camera.

### 2.7. Cytokine Analysis by Real-Time PCR

Mice skin was immediately frozen in liquid nitrogen and kept at −70°C until use. For the assay, skin was homogenized with Ultra-Turrax T10 (IKA labortechnik, Seoul, Korea) and RNA extraction was performed using TRIzol (Invitrogen life technologies, NY, USA). RNA content was measured using the NanoDrop ND-1000 spectrophotometer (NanoDrop Technologies Inc). 1 *μ*g of total cellular RNA from each sample was reverse transcribed using cDNA synthesis kit (TaKaRa, Japan). Quantitative PCR was performed using SYBR green iMaster and a LightCycler 480 (Roche, Switzerland). Primers for murin IL-4, IL-13, IL-17, and GAPDH are shown in Table 1. 

### 2.8. Plasma IgE Measurements

Total IgE levels in plasma were determined by sandwich ELISA using the BD PharMingen mouse IgE ELISA set. Briefly, plates were coated with capture antibody in ELISA coating buffer (sigma-aldrich) and incubated overnight at 4°C. Plates were washed with PBS-Tween 20 (0.05%) and subsequently blocked (10% FBS in PBS) for 1 h at 20°C. Serial dilutions of standard antigen or sample in dilution buffer (10% FBS in PBS) were added to the plates and plates were incubated for 2 h at 20°C. After washing, biotin-conjugated anti-mouse IgE and SAv-HRP (streptavidin-horseradish peroxidase conjugate) were added to the plates and plates were incubated for 1 h at 20°C. Finally, tetramethylbenzidine (TMB) substrate solution was added to the plates and after 15 min incubation in the dark, a 2N H_2_SO_4_ solution was added to stop the reaction. Optical densities were measured at 450 nm on an automated ELISA reader (Versa Max, Molecular Devices, CA, USA). 

### 2.9. Statistical Analysis

All experiments were expressed as the means ± standard deviations (SD) of at least three separate tests. Student's *t*-test was used for single variable comparisons, and a *P* value < 0.05 was considered statistically significant.

## 3. Results

### 3.1. KM110329 Decreased Inflammatory Cells in the Blood

**Figure 2:**
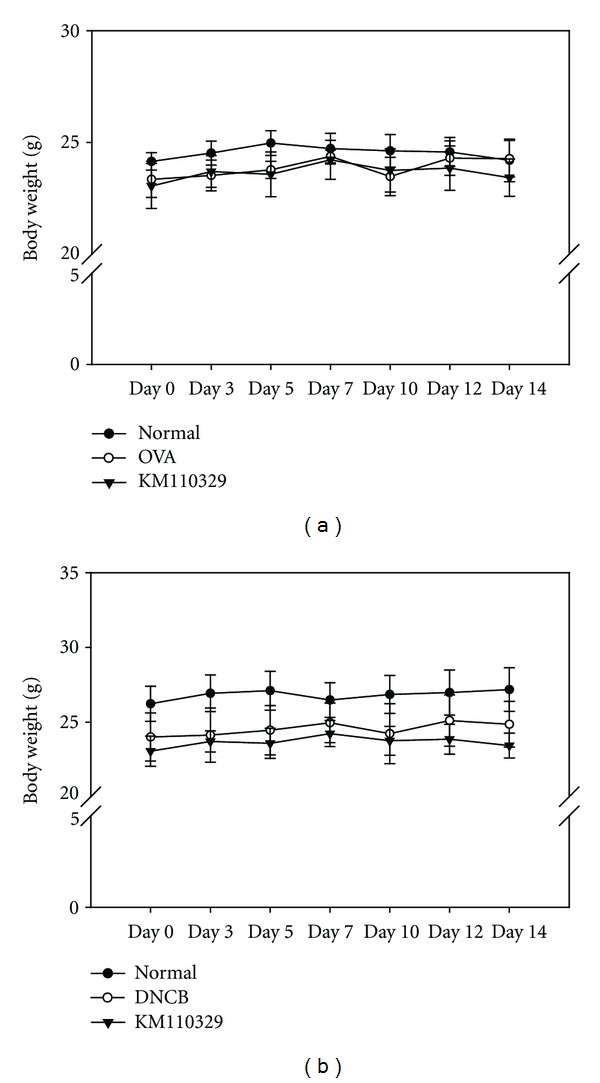
Changes in body weight during treatments with KM110329 in OVA- and DNCB-induced atopic dermatitis mice model. 100 ul of KM110329 (200 mg/mL) was applied on the mice back skin in the presence of OVA or DNCB for 2 weeks.

For the first attempt, we measured body weight of BALB/c mice treated with OVA or KM110329 to verify the toxicity of the drug. As seen in Figure 2, we found that KM110329 did not show any toxicity maintaining body weight. It is known that eosinophils, monocytes, neutrophils, and lymphocytes have been activated in atopic dermatitis patient's blood [17, 18]. Especially, eosinophils have been shown to be present in most patients with AD and correlated with the disease activity [19]. To investigate whether cutaneous KM110329 sensitization may decrease inflammatory cells, we measured leukocytes levels in cardiovascular blood samples using HEMAVET 950 hematology analyzer. Interestingly, Table 2 shows that KM110329 decreased significantly the numbers of eosinophils, monocytes, neutorphils, lymphocytes, and cells that contain metachromatic granules which probably represent mast cells.

### 3.2. Topical KM110329 Administration Decreases Infiltration of Inflammatory Cells into AD Skin Lesions

**Figure 3:**
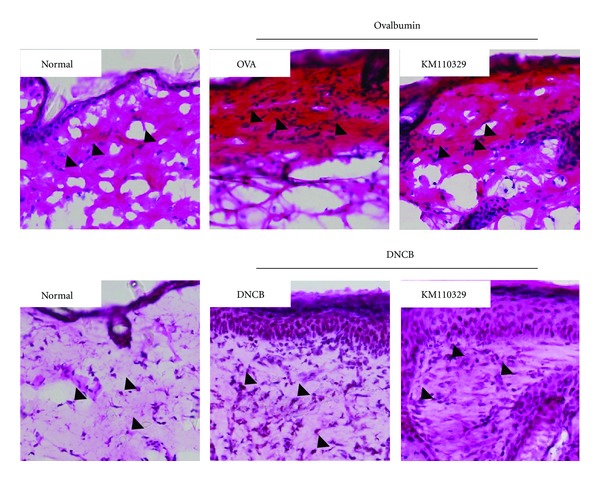
Histological features of AD-like skin lesions treated with KM110329. The skin sections were stained with hematoxylin and eosin. Inflammatory cells (arrows) infiltration into the dermis was measured after treatment with KM110329 in the presence of OVA or DNCB. Sections were evaluated using microscope at an original magnification of 400x.

**Figure 4:**
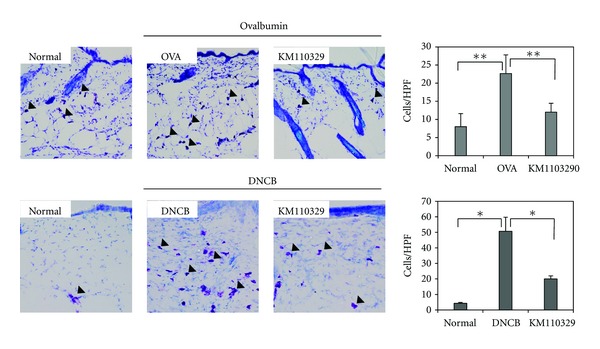
The measurement of mast cells number in AD-like skin lesions treated with KM110329. The skin sections were stained with toluidine blue for mast cells staining. Sections were evaluated using microscope at an original magnification of 400x. The data are presented as mean ± SEMs from four animals each group. *P < 0.05, **P < 0.01, and ***P < 0.001.

**Figure 5:**
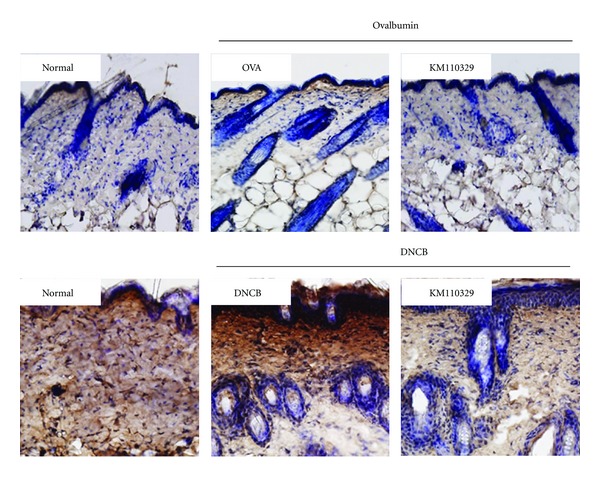
Distribution of CD3+ cells in skin samples. The skin sections were immunostained with CD3+ antibody. Epidermal CD3+ cells show a brown color. Sections were evaluated using microscope at an original magnification of 400x.

To determine whether KM110329 decreases infiltration of inflammatory cells into AD skin lesions, we performed H & E staining on the skin after topical administration of drugs. We observed infiltration of inflammatory cells into both the epidermis and dermis in control group (OVA, DNCB). Whereas, KM110329 decreased such infiltration of inflammatory cells into the skin (Figure 3). Next, we also performed toluidine blue staining for mast cell observation. Repeated cutaneous application of OVA and DNCB increased dermal mast cell number. However, this feature was significantly suppressed by KM110329 compared with control mice (Figure 4). In addition, OVA and DNCB increased numbers of CD3 + (Total T cells) while KM110329 decreased them in epidermal layer (Figure 5). 

### 3.3. KM110329 Administration Downregulates mRNA Expression of Cytokines

**Figure 6:**
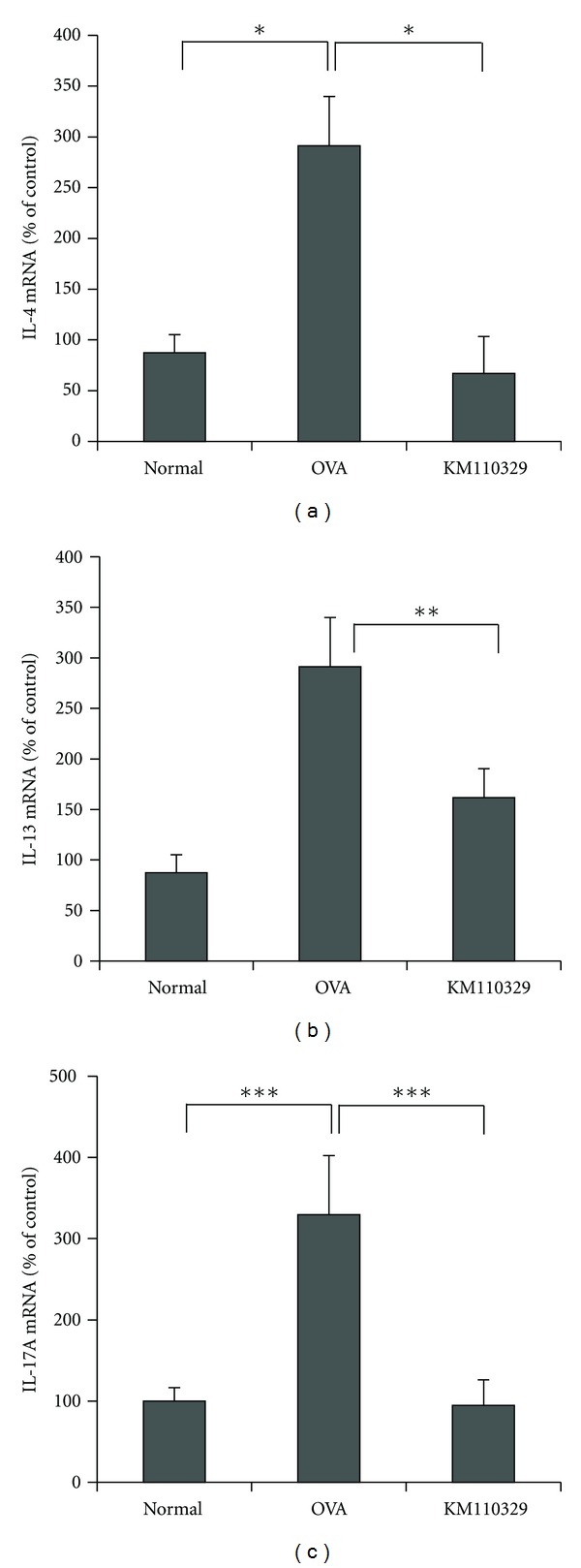
Effect of KM110329 on the expression of cytokines in skin tissue. IL-4, IL-13 and IL-17 mRNA expression levels were measured by real-time PCR. The columns and the error bars represent means ± SEMs (*n* = 4 mice/group). *P < 0.05, **P < 0.01, and ***P < 0.001.

 Th2 type cytokines are important in an acute phase of AD whereas mixed Th2/Th1 type inflammation is characteristic to a chronic phase of AD. Recently, a lineage of effector CD3+ T cells, that produces IL-17 and IL-17-producing T helper (T_H_-17) cells, has been identified. IL-17 mRNA and protein levels are elevated in patients with asthma and AD. As seen in Figure 6, we found that KM110329 decreased significantly relative mRNA expression of IL-4, IL-13, IFN-*γ*, and IL-17 induced by OVA and DNCB. 

### 3.4. KM110329 Administration Downregulates Serum IgE Concentration

**Figure 7:**
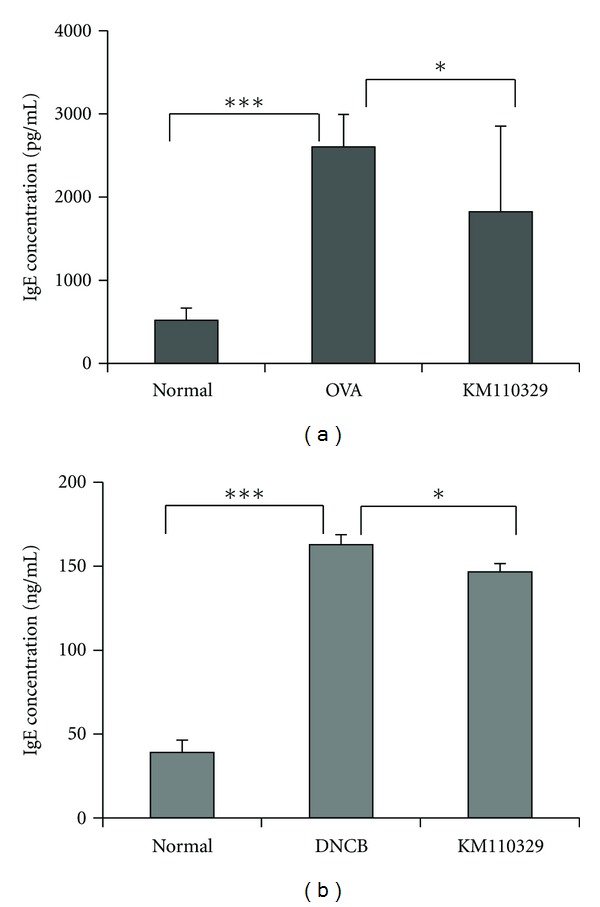
Measurement of Plasma IgE level. Total IgE level was determinated by ELISA. The columns and the error bars represent means ± SEMs (*n* = 4 mice/group). *P < 0.05, **P < 0.01, and ***P < 0.001.

Hyperproduction of IgE is a major characteristic of AD and patients with AD often exhibit elevated levels of total and allergen specific IgE antibodies (Abs) in their serum. Total IgE levels were elevated dramatically in OVA-treated group compared with normal group. However, increased serum IgE levels induced by OVA were significantly decreased by KM110329 treatment (Figure 7(a)). Also, increased serum IgE levels induced by DNCB were slightly decreased by KM110329 treatment (Figure 7(b)). 

## 4. Discussion

In this study, we investigated the effect of KM110329 on OVA- or DNCB-induced AD in BALB/c mice model. KM110329 significantly suppressed the leukocytes levels in blood samples. Blood analysis showed that topical administration of KM110329 markedly diminished the overexpression of leukocytes such as neutrophils, monocytes, lymphocytes, and eosinophils induced by OVA or DNCB. Histological analyses demonstrated that the infiltration of leukocytes into the skin lesions was decreased after KM110329 treatment. Topical administration of KM110329 prevented the aggravation of AD-like skin lesions. These effects of KM110329 on AD lesions suggest that KM110329 can be a potential alternative treatment for AD therapy.

The increase of eosinophils in the blood is the specific aspect of AD. The entrance of the eosinophils from the skin is related to C-C chemokines, eotaxin, and monocyte chemotactic 4 which are increased in AD [20]. The activation of eosinophils is induced by GM-CSF, IL-5, eotaxin and activated eosinophils release a variety of chemicals to cause inflammation and tissue injuries [21–23]. In our blood analysis, the count of eosinophils was decreased statistically significantly in KM110329-treated group resulting in the reduction of release of such chemicals which could induce skin diseases. This explains why the pharmacological drug treatment reduces the count of eosionphils below physiological drug levels.

Mast cells mediate inflammatory responses such as hypersensitivity and allergic reactions and the allergen cross-linking of surface IgE-dependent mast cells activation stimulates the degranulation and release of histamine, leukotrienes, proteases, prostaglandins, and cytokines. KM110329 is known to exert multiple biological effects such as anti-inflammatory activity and suppressive activity of Th2 immune response. Li et al., reported that *Houttuynia cordata *and *Rehmannia glutinosa* extracts suppressed compound 48/80-induced histamine release from mast cells *in vitro* [8, 24]. It was also reported that *Houttuynia cordata* inhibited the Th2 mediated inflammation through the downregulation of the production of Th2-cytokines in Jurkat T cells and the human mast cells [25].

Quantitative RT-PCR of the skin lesions also showed that topical KM110329 administration markedly diminished the mRNA level of Th2 cytokines such as IL-4 and IL-13 in the AD-like skin lesions. The production of proinflammatory cytokines such as IL-4 by epidermal cells has been identified as one of the main factors which mediates the initiation of AD [26]. AD is characterized by a predominant expression of Th2-type cytokines and associated with increased cellular infiltration at skin lesions, elevated circulating levels of IgE and eosinophilia [27]. Recent study reported that IL-17 cells infiltrated the papillary dermis of atopic eczema more markedly in the acute lesions than in the chronic lesions [4]. In our study, KM110329 downregulated the expression of IL-17 mRNA induced by OVA. Immunohistochemical study showed that KM110329 reduced increased number of CD3+ induced by OVA and DNCB. 

Recent data suggest that mast cells can contribute to eosinophil-mediated inflammatory responses. Mast cells-derived or T-cells-derived cytokines such as IL-5 and GM-CSF play an important role in eosinophil maturation and chemotaxis [28–30]. IL-5, TNF-*α*, and IL-2 also regulate chemotaxis, activation, and function of eosinophils. We found that KM110329 reduces mast cells (Figure 4) and inflammatory cytokines such as TNF-*α*, IL-6, and IL-8 by ELISA and RT-PCR (data not shown). It seems that the reduction of infiltration of mast cells is related to decrease of degranulation of mast cells and maturation of eosinophils suppressing the release of various inflammatory cytokines.

Our present study clearly demonstrates that KM110329 suppresses the progression of AD induced by OVA or DNBC. This suggests that KM110329 might be a useful candidate for the treatment of AD.

## Figures and Tables

**Table 1:** Primers.

Primer name	Sequence (5′–3′)
IL-4	Forward: AAGAACACCACAGAGAGTGAGCTC
	Reverse: TTTCAGTGTGGACTTGGACTC
IL-13	Forward: AGCATGGTATGGAGTGTGGACCTG
	Reverse: CAGTTGCTTTGTGTAGCTGAGCAG
IL-17A	Forward: AGCAAGAGATCCTGGTCCTGAA
	Reverse: CATCTTCTCGACCCTGAAAGTGA
GAPDH	Forward: GAGGGGCCATCCACAGTCTTC
	Reverse: CATCACCATCTTCCAGGAGCG

**Table 2:** Blood analysis. Blood leukocytes were analyzed using HEMAVET blood analysis system.

Cells	Normal range	OVA-induced group			DNCB-induced group		
		Normal	OVA	KM110329	Normal	DNCB	KM110329
WBC	1.8–10.7	3.77 ± 1.60	8.03 ± 2.09	4.25*± 1.78	4.69 ± 1.25	13.55 ± 2.60	1.71*± 0.30
Neutrophils	0.1–2.4	1.38 ± 0.50	2.25 ± 0.70	1.25*± 0.50	1.29 ± 0.28	4.79 ± 1.27	0.66*± 0.07
Lympocytes	0.9–9.3	2.54 ± 1.20	3.69 ± 0.90	2.56± 1.16	2.88–0.87	6.63 ± 1.13	0.82*± 0.22
Eosinophils	0.0–0.2	0.12 ± 0.10	0.43 ± 0.19	0.04*± 0.03	0.12 ± 0.06	0.87 ± 0.10	0.02*± 0.01
Monocytes	0.0–0.2	0.33 ± 0.08	0.60 ± 0.13	0.30*± 0.08	0.35 ± 0.08	0.97 ± 0.08	0.20*± 0.08

The columns and the error bars represent means ± SEMs (*n* = 4 mice/group). ^∗^ *P* < 0.05, **P < 0.01, and ***P < 0.001.
